# Intact cell mass spectrometry as a rapid and specific tool for the differentiation of toxic effects in cell-based ecotoxicological test systems

**DOI:** 10.1007/s00216-015-8937-2

**Published:** 2015-08-09

**Authors:** Sascha Liane Kober, Henriette Meyer-Alert, Desirée Grienitz, Henner Hollert, Marcus Frohme

**Affiliations:** Molecular Biotechnology and Functional Genomics, Technical University of Applied Sciences Wildau, Hochschulring 1, 15745 Wildau, Germany; Institute for Environmental Research (Biology V), Department of Ecosystem Analysis, RWTH Aachen University, Worringerweg 1, 52056 Aachen, Germany; Key Laboratory of Yangtze River Water Environment, Ministry of Education, College of Environmental Science and Engineering, Tongji University, 1239 Siping Road, Shanghai, 200092 China; College of Resources and Environmental Science, Chongqing University, Chongqing, 400030 China; School of Environment, Nanjing University, 163 Xianlin Ave., Nanjing, 210023 China

**Keywords:** RTL-W1, Intact cell mass spectrometry, MALDI, Acridine, β-Naphthoflavone, Copper sulfate

## Abstract

In the last few decades, MALDI-TOF MS has become a useful technique not only in proteomics, but also as a fast and specific tool for whole cell analysis through intact cell mass spectrometry (IC-MS). The present study evaluated IC-MS as a novel tool for the detection of distinct patterns that can be observed after exposure to a certain toxin or concentration by utilizing the eukaryotic fish cell line RTL-W1. Two different viability assays were performed to define the range for IC-MS investigations, each of which employing copper sulfate, acridine, and β-naphthoflavone (BNF) as model compounds for several classes of environmental toxins. The IC-MS of RTL-W1 cells revealed not only specific spectral patterns for the various toxins, but also that the concentration used had an effect on RTL-W1 profiles. After the exposure with copper sulfate and acridine, the spectra of RTL-W1 showed a significant increase of certain peaks in the higher mass range (*m*/*z* >7000), which is probably attributed to the apoptosis of RTL-W1. On the contrary, exposure to BNF showed a distinct change of ion abundances only in the lower mass range (*m*/*z* <7000). Furthermore, a set of mass peaks could be identified as a specific biomarker for a single toxin treatment, so IC-MS demonstrates a new method for the distinction of toxic effects in fish cells. Due to fast sample preparation and high throughput, IC-MS offers great potential for ecotoxicological studies to investigate cellular effects of different substances and complex environmental samples.

**Electronic supplementary material:**

The online version of this article (doi:10.1007/s00216-015-8937-2) contains supplementary material, which is available to authorized users.

**Graphical Abstract:**
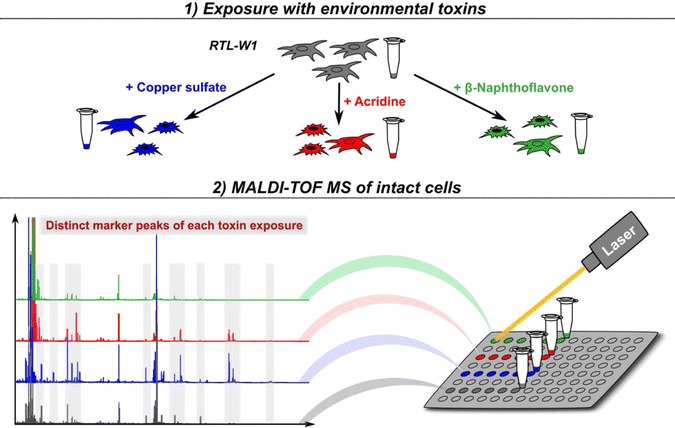
Use of intact cell MALDI-TOF mass spectrometry (IC-MS) to detect and differentiate toxic effects of environmental toxins in rainbow trout liver cell line RTL-W1

## Introduction

The investigation and risk evaluation of chemical pollutants are an important field of ecotoxicological studies [1, 2]. Xenobiotics enter our ecosystems in various ways, and most frequently, aquatic ecosystems are affected by contamination from effluents, such as those originating from agricultural pesticide runoff or tar oil pollution [3]. These effluents contain a slurry of chemicals that are incorporated via various human activities which commonly include substances such as metal compounds, polycyclic aromatic hydrocarbons (PAH), and heterocyclic aromatic compounds containing nitrogen, sulfur, or oxygen heteroatoms (NSO-Het) [4, 5]. Carrying out toxicity tests is essential to learn more about the effects of toxins on single organisms because of the direct consequences of some toxins on whole populations and ecology. For this purpose, cell cultures can strongly contribute to determine the mode of toxic action and also detect altered expression profiles [2]. The fish cell line RTL-W1 is most frequently used because of its ability to biotransform xenobiotics, i. e., chemicals and their metabolites can be detected subsequently [6, 7].

Viability assays are an easy and widely applied method to investigate the cytotoxicity of substances [8]. The concentration of the substance which causes 50 % of the maximum response (EC50) is used as a characteristic value for the evaluation of the cytotoxic effect of a substance [9].

In the 1990s, new applications of matrix-assisted laser desorption/ionization time of flight mass spectrometry (MALDI-TOF MS) were developed. For identification purposes, bacteria were placed directly from the agar plate onto the target without further purification steps [10]. The profiles, which were essentially fingerprints derived from proteins and peptides specific for each species, made it possible to identify bacterial cells through a comparison with deposited profiles collected in a database. Since then, the so-called intact cell mass spectrometry (IC-MS) became a fast, specific, and automatable analytical tool for clinical and environmental microbiology [11]. However, there have been only a few approaches that attempt to use this technique for eukaryotic cells. In 2006, Zhang et al. analyzed different mammalian cell lines that revealed specific IC-MS profiles [12]. Further investigations described direct characterization of primary glial cells [13], cell differentiation [11], cell death mechanisms [14], tissue-specific profiles of fish cells [15], phenotypic predictions [16], monitoring of drug treatments [17], or responses to toxic chemicals of mammalian cells [18]. Other fast evolving mass spectrometric methods for tissue and cell analysis include MALDI MS imaging [17, 19] and secondary ion mass spectrometry (SIMS), with focus on imaging of biological surfaces [19–21].

Since the identification of specifically expressed biomarkers is crucial for ecotoxicology [22], IC-MS could be a valuable tool for this field. The profiling of cells after the toxin exposure could reveal a set of distinct peaks that serve not only as biomarkers for toxic effects, but could also lead to the identification of toxins in complex environmental samples. For this purpose, the alteration of expression profiles of RTL-W1 cells after the exposure to three model substances belonging to different chemical classes were investigated in this study. Our aim was to find out if these changes are significant, as well as specific, for the toxin and the applied concentration. Therefore, the RTL-W1 cells were exposed to the metal compound copper sulfate, the NSO-Het acridine, and the PAH β-naphthoflavone (BNF). The viability studies, using two different assays, were carried out first to define the range for further IC-MS investigations.

## Materials and methods

### Reagents

All chemicals and solvents used were HPLC or analytical grade (p.a.). Acetonitrile (ACN), trifluoroacetic acid (TFA), sinapinic acid (SA), α-cyano-4-hydroxycinnamic acid (CHCA), and thiazolyl blue tetrazolium bromide (MTT) were obtained from Sigma (St. Louis, MO, USA).

Dimethylsulfoxide (DMSO; cell culture grade) was purchased from Neolab (Heidelberg, Germany), and phosphate buffered saline (PBS) was obtained from Gibco by Life Technologies (Darmstadt, Germany).

CellTiter-Blue® and CellTiter-Glo® Luminescent Cell Viability Assays as well as CytoTox 96® Non-Radioactive Cytotoxicity Assay were provided by Promega (Madison, WI, USA).

Milli-Q water (ddH_2_O, Millipore) was prepared in-house.

### Cell culture

In this study, RTL-W1, a liver cell line from rainbow trout (*Oncorhynchus mykiss*) was used. It was obtained from Drs. Niels C. Bols and Lucy Lee, University of Waterloo, Canada [6]. The cells were routinely grown according to the method detailed by Hinger et al. [23] at 20 °C in Leibovitz L15 medium (Biochrom, Berlin, Germany) supplemented with 10 % fetal calf serum (Biochrom), 5 mM l-alanyl-l-glutamin (Biochrom) and 1 % antibiotics (Gibco by Life Technologies). Cells were grown to approximately 90 % confluency before passaging for experimental use.

### Toxin exposure

Three toxins were used to induce cellular effects: copper sulfate (Sigma), acridine (Sigma), and β-naphthoflavone (BNF; Sigma).

Viability studies were performed in 96-well assay plates. Cells were seeded at a density of 20,000 viable cells/well and cultivated for approximately 24 h. Subsequently, medium was replaced with exposure medium. The exposure medium was either L15 without FBS and antibiotics for the investigation of acridine and BNF or L15/ex for copper sulfate. L15/ex was prepared according to Morton [24] and Leibovitz [25] and contained only physiological salts, galactose, and sodium pyruvate of L15. A 1-μL aliquot of a toxin stock solution was added, and the cells were incubated for 24 h based on the methods of Dayeh et al. [26]. Copper sulfate stock solutions were diluted using L15/ex, whereas DMSO was used for acridine and BNF. With respect to prior experiments, the final DMSO concentration in all wells was limited to a maximal content of 1 %. For all experiments, control treatments with DMSO and toxin treated cells were run in parallel. Wells without cells were prepared with medium and DMSO if necessary for background correction.

Exposures of RTL-W1 cells with acridine were performed with a gastight cover sheet to limit evaporation of the volatile substance.

For IC-MS studies, cells were seeded in T25 cell culture flasks with a total amount of 1.7 × 10^6^ cells. Three to five concentrations for each toxin were chosen according to the results of the EC50 calculation, representing comparable viability declines for each series of tests. Toxin exposure was performed according to the viability studies.

### Viability assays and EC50 calculation

After 24 h, the cultures were assayed for viability according to the manufacturer’s instructions (Promega). After a final incubation time of 4 h for CellTiter-Blue® Viability Assay, fluorescence was recorded at 540/595 nm on Tecan infinite 200 Pro microtiter plate reader (Tecan, Männedorf, Switzerland). The integration time for luminescence detection (CellTiter-Glo® Luminescent Cell Viability Assays) on Tecan GENios Plus microtiter plate reader (Tecan) was 500 ms per well.

Statistical analysis and EC50 calculations were performed using GraphPad Prism (Graphpad Software, San Diego, USA). Data were baseline corrected and normalized according to the control treatments. EC50 values were calculated with a sigmoid dose–response fit and automatic outlier detection.

### Intact cell mass spectrometry

After 24 h of toxin exposure, cells were harvested and washed with PBS at 2000×*g*, 4 °C for 5 min (Eppendorf 5424, Hamburg, Germany) based on the method detailed by Dong et al. [14]. The total number of cells was calculated using an automated cell counter (Cedex XS Analyzer, Roche Innovatis, Bielefeld, Germany). The cell pellets were stored at −20 °C until IC-MS analysis.

According to the calculated cell number, stock solutions with 5 × 10^4^ cells per μL as well as further dilutions of 2 × 10^4^ and 1 × 10^4^ cells per μL were prepared with PBS und stored on ice prior to IC-MS.

Samples and matrix solutions were deposited on the MALDI-TOF MS target layer by layer and successively air dried. First, a thin layer of saturated SA in ethanol was applied to an Opti-TOF 384 well insert (Kratos Analytical, Manchester, UK), followed by 0.5 μL of the sample solution and, finally, covered with 0.5 μL of SA matrix (38 mg/ml in water/ACN/TFA 40/60/0.2 (*v/v/v*)) which was presented by Munteanu et al. [11]. All sample dilutions were spotted in duplicate.

MALDI-TOF mass spectra were obtained using an Axima Confidence mass spectrometer (Shimadzu Biotech, Manchester, UK) equipped with a 337-nm nitrogen laser. The filter for regulation of the laser firing power was set to 65. To generate representative profiles, a total of 600 laser shots were accumulated and averaged for each sample. Protein mass fingerprints were obtained in the linear, positive mode at a maximal laser repetition rate of 50 Hz within a mass range of 2000 to 20,000 Da.

MALDI-TOF MS data processing was performed using the LaunchpadTM v. 2.9 software (Shimadzu Biotech). A set of 10 peaks that occurred in the majority of spectra and were evenly distributed across the whole mass range were used for internal calibration.

For peak acquisition, an average smoothing method was performed with a smoothing filter width of 50 channels and a baseline filter width of 100 channels. Peak detection was performed using mMass, an open source mass spectrometry tool (www.mmass.org). The criteria included a S/N ratio of 3 and an absolute ion abundance of 200 mV. For each spotted sample, a list of spectrum peaks was generated and analyzed statistically. The masses of equally treated samples were matched and means and standard deviations of ion abundances calculated. Statistically significant changes compared to the control treatment were investigated using GraphPad Prism. For principal component analysis (PCA), relative ion abundances were calculated and data normalized according to the control treatments. It was carried out using the free statistical computing software R (www.r-project.org). The Grubbs’ test was conducted prior to exclude significant outliers.

## Results

### Viability assays and EC50 calculation

Prior to the cytotoxic studies of the model compounds copper sulfate, acridine, and BNF, different commercial viability assays were compared for use with RTL-W1: the conventional MTT assay, the CellTiter-Blue® and CellTiter-Glo® Luminescent Cell Viability Assays as well as CytoTox 96® Non-Radioactive Cytotoxicity Assay. The CellTiter-Blue® and CellTiter-Glo® showed the best performance, with good signal linearity up to 40,000 cells per well and low signal variability (see Electronic Supplementary Material (ESM) Fig. S1). For that purpose, both assays were used for the determination of viability and EC50 values. All experiments included microscopic investigations with a BZ-9000E (Keyence, Neu-Isenburg, Germany) to evaluate morphologic alterations 24 h after toxin exposure. For all toxin treatments, similar effects on RTL-W1 cells could be observed, as rounding and detachment of the cells occurred with an increase of the concentration (data not shown).

**Fig. 1:**
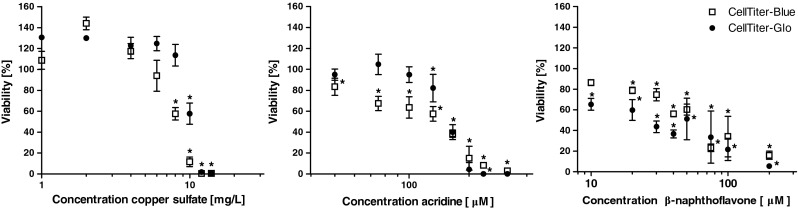
Cytotoxic effects of copper sulfate, acridine, and BNF in RTL-W1 after an exposure time of 24 h. Data are shown as means and standard deviations of four independent experiments in percentages relative to the controls for the CellTiter-Blue® and CellTiter-Glo® viability assays. *Asterisks* indicate significant decreases in cell viability compared to the controls (one-way ANOVA followed by Dunnett’s test)

In all experimental series, the results differed between the CellTiter-Blue® and CellTiter-Glo® viability assays that were obtained 24 h after toxin treatment. Whereas the CellTiter-Blue® assay showed a faster viability decrease of RTL-W1 for copper sulfate and acridine, the CellTiter-Glo® assay proved to be more sensitive for low concentrations of BNF (Fig. 1).

Low concentrations of copper sulfate (1 to 4 mg/L) led to a partly significant increase in viability according to the control treatments. Significant declines in viability were observed for concentrations ranging from 8 to 10 mg/L copper sulfate (Fig. 1a). EC50 values of 8.23 and 10.08 mg/L reflected these results (Table 1).

**Table 1:** EC50 values and 95 % confidence intervals for cytotoxicity of copper sulfate, acridine and BNF in RTL-W1 cells after 24 h exposures using CellTiter-Blue® and CellTiter-Glo® viability assays

	CellTiter-Blue®, 24 h		CellTiter-Glo®, 24 h	
	95 % conf. interval EC50	EC50	95 % conf. interval EC50	EC50
Copper sulfate [mg/L]	7.58–8.90	8.23	7.62–13.33	10.08
Acridine [μM]	106.17–124.35	114.90	140.29–146.96	143.58
BNF [μM]	46.08–60.08	52.62	20.95–28.36	24.37

The exposure with acridine resulted in a significant decrease in viability starting with concentrations of 50 μM and continuing up to 125 μM. The viability decline investigated with CellTiter-Blue® is almost constant between 50 and 250 μM acridine, whereas a distinct drop of the signal in the CellTiter-Glo® assays is beyond 125 μM, resembling a sigmoid curve (Fig. 1b). As with copper sulfate, the EC50 value obtained with the CellTiter-Blue® viability assay (114.90 μM) is lower than the value of the CellTiter-Glo® viability assay (143.58 μM) (Table 1).

Cells exposed to BNF showed a significant decrease in viability signals with concentrations of 10 and 20 μM. Beginning at 50 μM, BNF viability signals showed a higher standard deviation (Fig. 1c). In contrast to the other model compounds, the CellTiter-Glo® assay resulted in a remarkably lower EC50 value of 24.37 μM compared to 52.62 μM of CellTiter-Blue® assay (Table 1).

### Intact cell mass spectrometry: RTL-W1 profiles after 24 h exposures

To obtain remarkable and reproducible profiles of the RTL-W1 cells, the IC-MS method was optimized first. Parameters such as the choice of the matrix, the spotted cell number, and the pretreatment (washing) of the cells after toxin treatment were investigated. The comparison of SA and CHCA matrices showed the most distinct profiles with a high concentrated SA matrix according to Munteanu et al. [11] (ESM Fig. S2). Washing buffer PBS proved to be superior to 150 mM ammonium acetate, which was recommended by Hanrieder et al. [13] (ESM Fig. S3). For spotting on the MALDI target, a cell number of at least 2500 cells was necessary to acquire distinct protein profiles. In the IC-MS toxin studies, 5000, 10,000, and 25,000 cells were spotted. A number of 10,000 cells proved to be most efficient for MALDI-TOF mass spectrometry and was consequently used for interpretation of data.

As a result of the viability tests, different toxin concentrations around the EC50 were chosen for IC-MS measurements in RTL-W1. Copper sulfate exposures were performed with 2, 4, 6, 9, and 12 mg/L while acridine was used in concentrations of 50, 100, and 150 μM. Finally, cells were treated with 10, 30, and 50 μM BNF. Control treatments were performed with the highest DMSO concentration used in the appropriate experimental series.

**Fig. 2:**
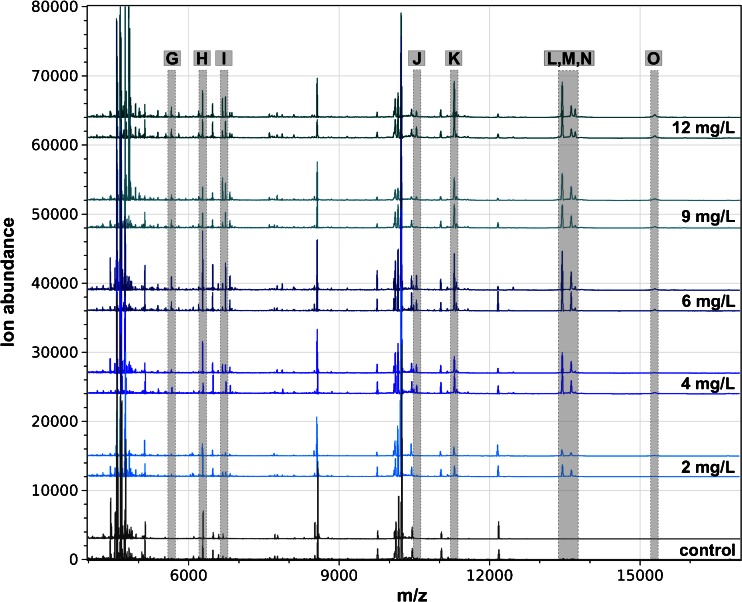
Intact cell MALDI-TOF MS analysis of RTL-W1 exposed to various concentrations of copper sulfate. Mass spectra are shown as biological duplicates for each concentration in the mass range from  *m*/*z* 4000 to 17,000 and shifted to allow discrimination of interspectral differences. Distinctive peaks are shaded in *gray* and named from G to O

**Fig. 3:**
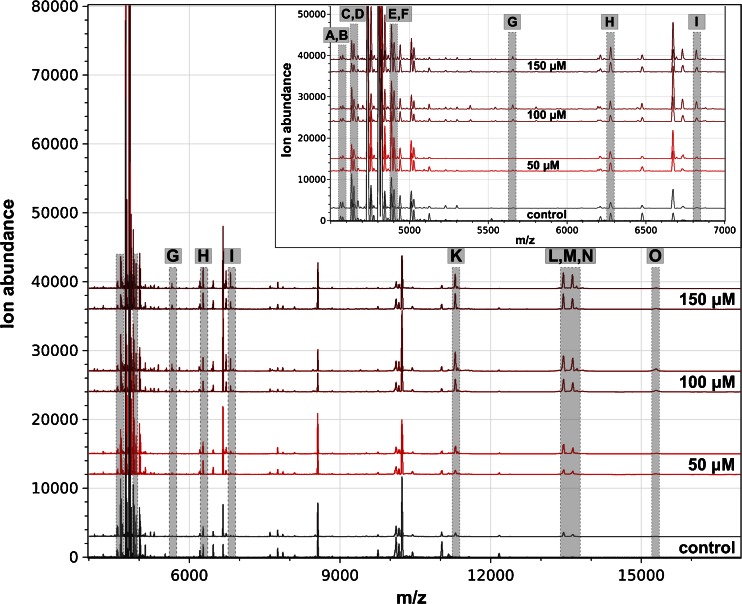
Intact cell MALDI-TOF MS analysis of RTL-W1 exposed to various concentrations of acridine. Mass spectra are shown as biological duplicates for each concentration in the mass range from  *m*/*z* 4000 to 17,000 and  *m*/*z* 4500 to 7000 (*insets*), and shifted to allow discrimination of interspectral differences. Distinctive peaks are shaded in *gray* and named from A to O

**Fig. 4:**
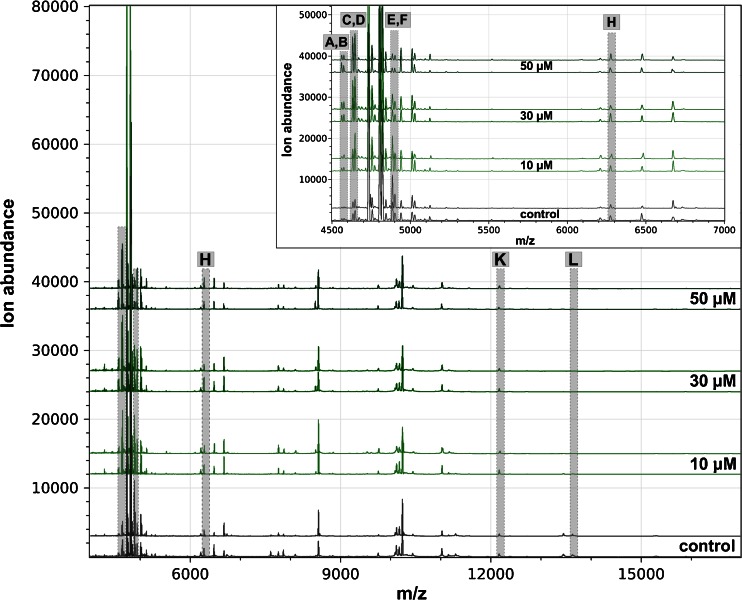
Intact cell MALDI-TOF MS analysis of RTL-W1 exposed to various concentrations of BNF. Mass spectra are shown as biological duplicates for each concentration in the mass range from  *m*/*z* 4000 to 17,000 and  *m*/*z* 4500 to 7000 (*insets*), and shifted to allow discrimination of interspectral differences. Distinctive peaks are shaded in *gray* and named from A to L

In the range of *m*/*z* 4000 to 17,000, several peaks were observed that changed significantly as a result of the toxin treatment. For all toxins, the effects are most noticeable for 15 peaks that were labeled from A to O, whereas distinct signals were highlighted in gray for every single toxin treatment (Figs. 2, 3, and 4).

**Fig. 5:**
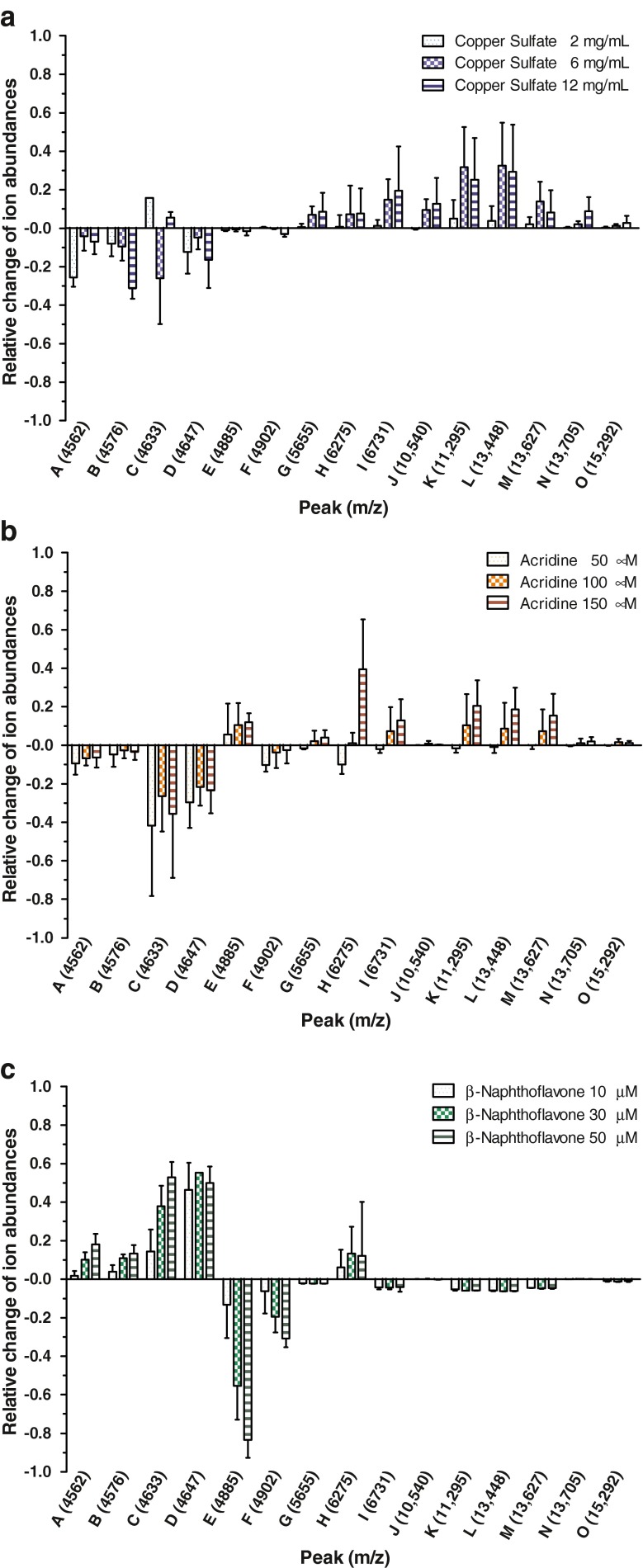
Relative change of ion abundances of spectra of RTL-W1 exposed to various concentrations of copper sulfate (**a**), acridine (**b**), and BNF (**c**) compared to the controls. Data are shown as means and standard deviations of six independent experiments

The exposure of RTL-W1 to copper sulfate led up to 100 signals with a higher ion abundance of 200 mV. The statistical analysis revealed nine peaks that showed a gradual increase after the treatment of RTL-W1 with different copper sulfate concentrations (Fig. 2). Specifically, peaks I (*m*/*z* 6731), K (*m*/*z* 11,295), L (*m*/*z* 13,448), M (*m*/*z* 13,627), and N (*m*/*z* 13,705) showed remarkable changes. Even small concentrations of 2 and 4 mg/L copper sulfate led to an increase in some signals. That effect increased with higher concentrations and was even expanded by more peaks (Fig. 2). To compare the ion abundances of different concentrations directly, we normalized the relative ion abundances of the 15 peaks according to the controls and plotted the change for all peaks and substances (Fig. 5). There is a distinct difference between the low concentration (2 mg/mL) and the high concentrations of copper sulfate (6 and 12 mg/mL). For some peaks (G, I, N, and O), the change of ion abundances increases between 6 and 12 mg/mL (Fig. 5a).

The treatment with acridine also led to a change in several significant peaks as a consequence of the toxin exposure (Fig. 3). Besides the increase in peaks K, L, and M, which were already found in the copper sulfate studies, some peaks in the lower mass range changed significantly. In contrast to the peaks already mentioned, the signals A (*m*/*z* 4562), C (*m*/*z* 4633), and D (*m*/*z* 4647) showed a decrease following the acridine treatment of RTL-W1. On the other hand, ion abundances of the peaks E (*m*/*z* 4885) and G (*m*/*z* 5655) were slightly increased. Some of those peaks show a clear concentration dependency (Fig. 5b).

Finally, the incubation of RTL-W1 with BNF resulted in completely different profiles. Almost no signals were observed in the higher mass range as a result of the toxin treatment (peaks K, and L in Fig. 4). In the lower mass range, the peaks A, B (*m*/*z* 4576), C, and D (*m*/*z* 4647) revealed increasing ion abundances with higher BNF concentrations, whereas the peaks E and F decreased significantly (Fig. 5c).

### Intact cell mass spectrometry: comparison of toxin exposures

RTL-W1 profiles after the treatment with 9 mg/L copper sulfate, 150 μM acridine, and 50 μM BNF were compared directly because they resulted in a similar viability impairment of roughly 50 %.

The 15 distinctive peaks between *m*/*z* 4562 and *m*/*z* 15,292 were analyzed for each toxin treatment: The arithmetic mean of the ion abundances was calculated from the six replicates and the direction of deviation from the control treatment was determined and tested for significance (paired two-sample *t* test) (Table 2).

**Table 2:** Distinctive peaks of IC-MS of RTL-W1 24 h after exposure to copper sulfate (9 mg/L), acridine (150 μM), and BNF (50 μM)

Peak		Significant changes		
Label [/]	Mass [*m*/*z*]	Copper sulfate 9 mg/L [/]	Acridine 150 μM [/]	BNF 50 μM [/]
A	4562	–	↓ **	↑ *
B	4576	–	–	↑ *
C	4633	↑ **	↓ **	↑ *
D	4647	–	↓ *	↑ *
E	4885	–	–	↓ **
F	4902	–	↑ **	↓ **
G	5655	↑ *	–	–
H	6275	–	–	↑ *
I	6731	↑ **	–	–
J	10,540	↑ *	–	–
K	11,295	↑ **	↑ *	↓ *
L	13,448	↑ **	↑ *	↓ *
M	13,627	–	↑ *	–
N	13,705	↑ *	–	–
O	15,292	↑ *	–	–

*p < 0.05, **p < 0.01 indicate significant increase (upwards arrow) or decrease (downwards arrow) of ion abundance compared to the control, paired two-sample *t* test, *n* = 6

In the lower mass range, mainly acridine and BNF revealed changes in the ion abundances in the expression profiles of RTL-W1. Whereas ion abundances decreased after acridine exposure, the same signals increased significantly after BNF treatment. Cells treated with copper sulfate showed mainly an increase of signals in the higher mass range (peaks I,J, K, L, M, N, and O). All toxin profiles have the changes of the peaks C, K, and L in common, although the types of deviation varied. The signals B, E, H (BNF) as well as G, I, J, N, O (copper sulfate), and M (acridine) were specifically altered for one toxin.

**Fig. 6:**
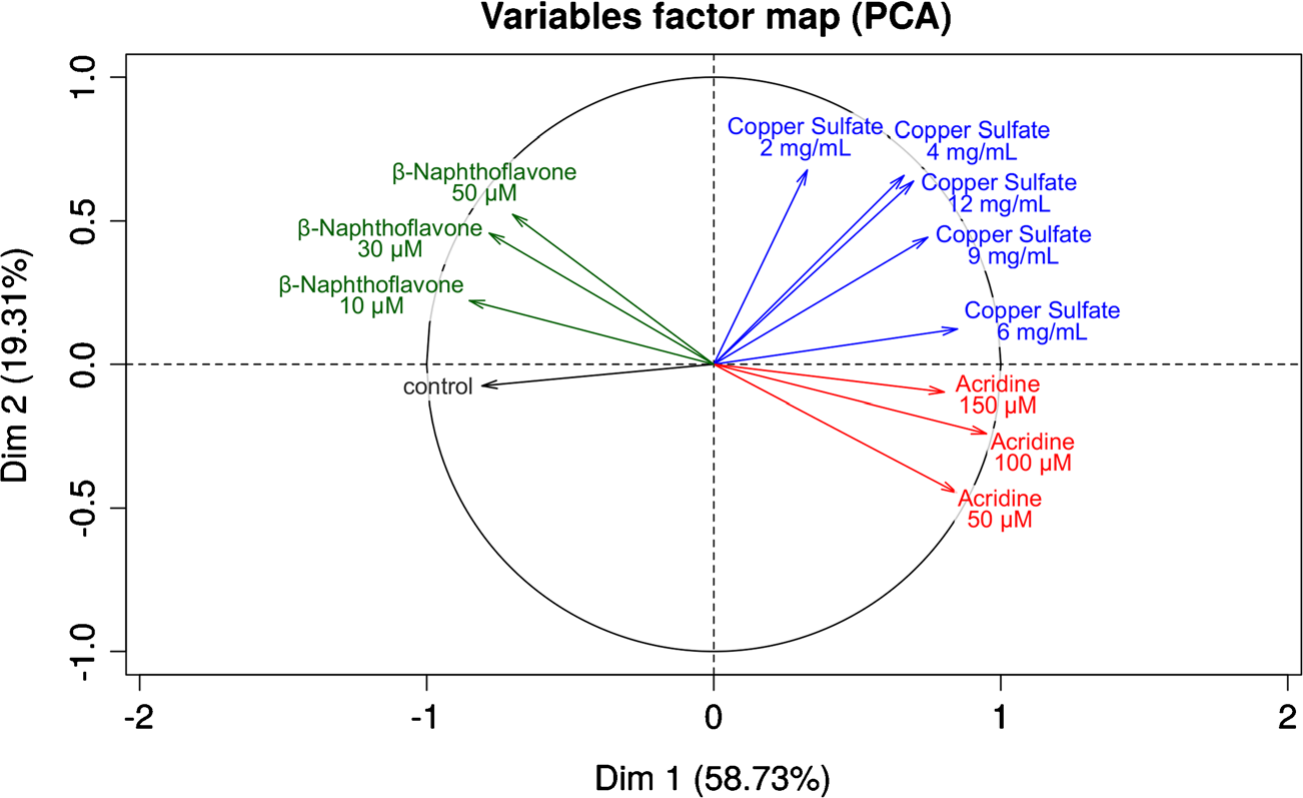
Principal component analysis (PCA) of the peak data obtained from Fig. 5. The change of ion abundances for each individual peak (A to O) and toxin concentration were analyzed and plotted as variables factor map

To prove the toxin specificity of the profiles, we conducted principal component analysis (PCA) for the identified marker peaks (Fig. 6). For each peak, the relative change of ion abundance compared to the control was investigated for the different substances and concentrations. The main data variance (59 %) is described by principal component 1 (PC1), whereas PC2 stands for 19 % of the data variance. For the representation of the control, the means of all control treatments were used for PCA. The arrows representing the different toxin and control treatments are located in different quarters of the PCA plot, which indicates that the peak changes are specific. The variance representation of the principal components is furthermore a hint that the changes of peak patterns of copper sulfate and acridine have some changes in common. Besides, a concentration dependent shift for BNF and acridine variables can be observed.

## Discussion

The present study shows a novel approach to measure toxicological effects in fish cells. This is not only specific for certain toxins, but also reveals a concentration-dependent performance. The use of whole cells for MALDI-TOF mass spectrometry is well established for identification of bacteria [10]. In recent years, this technology has been increasingly deployed for applications to create characteristic profiles of eukaryotic cell lines [11–18]. The use of IC-MS to produce specific expression profiles of fish cells and identify toxins through their specific patterns as a biomarker is a new approach in ecotoxicology.

For that purpose, we investigated the effects of three toxins belonging to three different chemical classes on the rainbow trout liver cell line RTL-W1: the metal compound copper sulfate, the NSO heterocyclic compound acridine, and the PAH compound BNF.

Firstly, a concentration range of each toxin was determined with different viability assays. The CellTiter-Blue® and CellTiter-Glo® record the cell viability according to the metabolic impairment in consequence to the toxin treatment. Whereas the CellTiter-Blue® measures the redox activity and hence the content of NADPH and NADH [27], the CellTiter-Glo® focuses on kinase activity and detects ATP levels in the cell [28]. Two different cell parameters are considered providing further information about the mechanisms of cellular actions affected by the respective substance [29]. The chosen cell number of 20,000 cells per well in a 96-well microtiter plate format is in accordance with the cell number usually applied in viability assays with fish cells [26, 30, 31]. The final DMSO concentration never exceeded 1 % (*v*/*v*) because in former studies, this concentration revealed first cytotoxic effects [32]. As acridine is a volatile substance, a gastight cover sheet was used for viability studies (cf. [23, 33]). Nevertheless, a loss of substance over time should be considered due to adsorption onto the well surface or precipitation. Peddinghaus et al. and Hinger et al. reported a loss of substance although a gastight cover sheet was used [5, 23].

The exposure of RTL-W1 with copper sulfate and acridine led to lower EC50 values for CellTiter-Blue® than CellTiter-Glo® viability assays. This could be related to a greater impairment of the redox activity of the cells. Primarily, cellular reductases, particularly diaphorases, are responsible for the conversion of resazurin into resorufin in the CellTiter-Blue® viability assay [27]. In contrast to that, the CellTiter-Glo® viability assay detects the amount of ATP in the cells via a luciferase reaction. As ATP is a common substrate of kinases, its detection delivers information about the kinase activity in the cell [28]. Consequently, the exposure with copper sulfate and acridine affected the reductases prior to the ATP content of the cell. The higher signals of low copper sulfate concentrations in contrast to the control treatment could be related to a higher metabolic activity as a response to the toxin exposure.

The molecular mechanisms of action for copper sulfate are still unknown. Nevertheless, a study with RTgill-W1 cells proved a significant increase in reactive oxygen species (ROS), as well as the induction of DNA strand breaks, as a consequence of the copper sulfate exposure [34].

The cellular effects of acridine could have a similar background. However, only few in vitro viability studies examined NSO-HETs so far and further investigations are crucial [23, 33].

The third substance, BNF, showed different results than copper sulfate and acridine. The CellTiter-Glo® viability assay was markedly more sensitive than the detection of reductases activity. This leads to the conclusion that the mode of action is unlike copper sulfate or acridine. Already, a concentration of 10 μM BNF resulted in a signal decrease. It can be assumed that a distinct EROD activity is induced by this concentration [30] and hence an increased kinase activity. Possibly not only the cytotoxic effect of BNF but also the increased ATP consumption consequent to the enzyme induction attributed to the decreased luminescence signals.

High concentrations of BNF resulted in precipitation in the culture medium due to its low solubility. Consequently, the effective concentration of BNF could be lower and should be considered for the estimation of its viability impairment or induction potency.

The calculated EC50 values for RTL-W1 cells with copper sulfate were a bit higher than literature values, e.g., 3.69 mg/L (redox activity) and 6.56 mg/L (membrane integrity) [26]. However, several test series confirmed the concentration range in this work and the influence of different assays should be considered. An EC20 of 40.9 mg/L (228 μM) was recorded for acridine with neutral red retention assay in preparation for micronucleus assays with RTL-W1 [33]. This indicates a much higher EC50 value than presented in this work, which is also ascribed to the different detection principle of the viability assays. For BNF, no reference values could be found, but likewise, several test series were performed. Therefore, the listed values are an indication for further investigations.

The performance of viability studies resulted in concentration ranges that revealed cytotoxic effects of copper sulfate, acridine, and BNF on RTL-W1 cells and were a starting point for IC-MS investigations. For that purpose, concentrations were chosen that resulted in a viability decline of roughly 10, 40, and 60 %. All three model compounds induced profile variations of RTL-W1. Furthermore, the six independent measurements for each concentration revealed similar profile changes. At least seven peaks were detected that changed significantly through the toxin exposure (acridine). For copper sulfate (eight peaks) and BNF (nine peaks), even more signals showed significant alterations. Particularly striking is the progressive increase of ion abundances at *m*/*z* 6731, 11,295, 13,448, and 13,627 as a result of copper sulfate and acridine exposure of RTL-W1. Those peaks were also increased in a study with preapoptotic substances with HeLa cells and other mammalian cell lines [14]. Microscopic studies support the assumption that the alteration of those peaks is also related to apoptosis of the fish cell line RTL-W1. Even for the lowest concentration of 2 mg/L copper sulfate, the IC-MS showed an apoptotic behavior, whereas the viability studies did not suggest a viability decline for this concentration. Cells treated with acridine showed additional variations in the low mass range. The signals at *m*/*z* 4562, 4576, 4633, and 4647 decreased notably compared to the control treatment. In contrast, the BNF-treated cells showed increased ion abundances in the lower mass range, whereas a significant decrease in the higher mass range could be observed.

Consequently, the results of the IC-MS reveal a different molecular mode of action when comparing BNF to acridine and copper sulfate likewise, which was confirmed by PCA. Microscopic investigations of BNF treated cells did not show typical signs of necrosis (e.g., cellular swelling) [35]. However, results of several studies indicate that the various cell death pathways (apoptosis, necrosis, and autophagy) correlate more than previously thought [36]. Hence, the information of microscopy and IC-MS are not sufficient to make a final statement on cell death of RTL-W1 after treatment with BNF.

The IC-MS of cells that were treated with mono-substances or complex samples offer a great potential for typing cytotoxic effects. A characteristic set of peaks could be used as a biomarker to identify different ecotoxicologically relevant substances in soil, sediment, or groundwater samples.

As of now, there have been few attempts made to identify the intracellular mechanisms that are correlated with altered peaks of IC-MS profiles. In particular, the identification of certain proteins or peptides behind the peaks in cell profiles is a desirable objective [12].

Bergquist et al. found altered ion abundances between *m*/*z* 4000 and 18,000 in profiles of the rat cell line PC 12 that could be related to stimulation with a nerve-growth factor [37]. Hanrieder et al. used SDS-PAGE and ESI-MS to identify proteins of glial cells that showed up in the IC-MS profiles [13]. Zhang et al. were able to identify a few proteins in the lower mass range that are either common between different mammalian cell lines or appeared specifically [12]. Some of the peaks that were found in this work were also mentioned by Zhang, Bergquist and Hanrieder, e.g., *m*/*z* 5655, 6275, and 11,295. Most notably, the latter was found in all three studies and identified as a modified form of histone 4 [13]. Furthermore, two forms of the structuring protein thymosin were identified in the mass range below 5000 Da. Since the peaks *m*/*z* 4885 and 4902 of RTL-W1 were significantly lower after the exposure with BNF, this could be related to changes in the organization of the cytoskeleton [38]. Similarly, other peaks could stand for certain molecular changes that were caused by the toxin exposure. However, the identification of peptides and proteins was not attempted in this work. Besides, one has to keep in mind that no prior purification steps were performed and hence, the peaks most likely do not refer to single proteins. Additionally, posttranslational modifications, e.g., glycosylation and phosphorylation, have to be considered, and thus only the mass is insufficient for protein identification [37].

### Conclusion

This study outlines an initial attempt to identify toxic effects on RTL-W1 cells by IC-MS. The changes of certain mass peaks were not only toxin specific, but also depended on the used concentration. Consequently, a set of peaks could be identified as a biomarker for a certain toxin or chemical class. Hence, IC-MS could be a valuable tool for the profiling of toxic effects and risk assessment of ecosystems. Since it is a fast, easy-to-use, and automatable method, it is suited for the high throughput of environmental samples as well. The fast identification of the substances that are responsible for the contamination could prevent further pollution or initiate environmental remediation. However, there are still several steps necessary to evaluate complex samples that could eventually identify molecular mechanisms that are related to peak changes in IC-MS. Other metal compounds, PAH, NSO-Hets, and structural analogs, have to be analyzed to look for specific or common changes of spectral patterns. As for now, it is not possible to quantify the toxic effects with the presented method, but new matrices or internal standards may offer the opportunity to directly relate toxin concentration and ion abundances. Further research is needed to identify the potential whether the developed method could be used as an effect-based tool in the context of the different monitoring programs linking chemical and ecological status assessment within the water framework directive [39].

## Electronic supplementary material

ESM 1 (PDF 671 kb)
