# Supplementary material for: Intact cell mass spectrometry as a rapid and specific tool for the differentiation of toxic effects in cell-based ecotoxicological test systems
Source: Anal Bioanal Chem. 2015 Aug 9;407(25):7721–31. doi: 10.1007/s00216-015-8937-2 (PMC4575386; doi:10.1007/s00216-015-8937-2)
Supplement: Supplementary file 1 — (PDF 671 kb) [file 216_2015_8937_MOESM1_ESM.pdf]

**Analytical and Bioanalytical Chemistry**

**Electronic Supplementary Material**

# Intact cell mass spectrometry as a rapid and specific tool for the differentiation of toxic effects in cell based ecotoxicological test systems

Sascha Liane Kober, Henriette Meyer-Alert, Desirée Grienitz, Henner Hollert, Marcus Frohme

## 1) Viability Assays

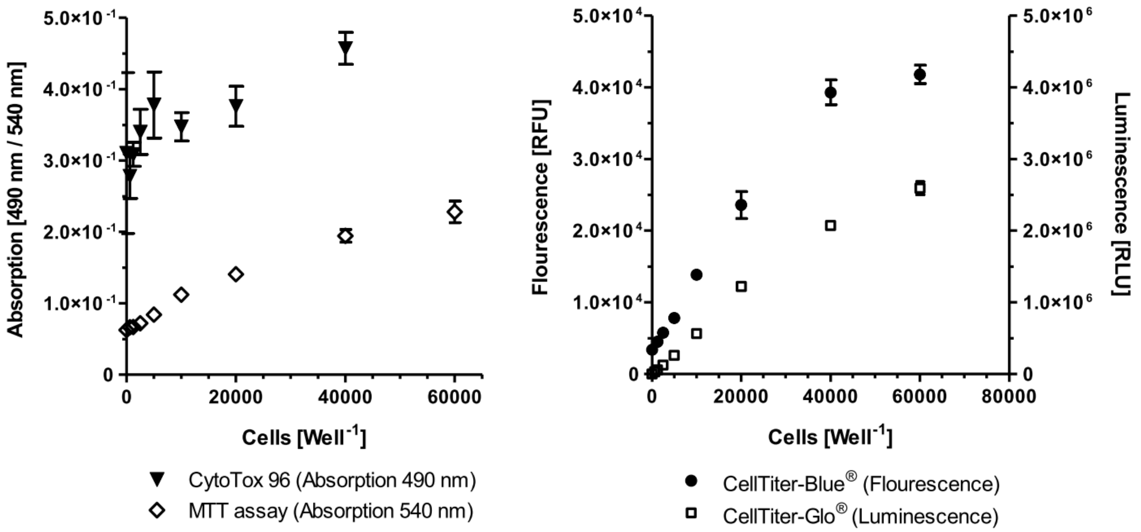


**Fig. S1** Sensitivity of different viability assays 24 h after seeding of different cell numbers: absorption signals of the conventional MTT assay and CytoTox 96® Non-Radioacitve Assay (Promega) showed low intensities and higher variation whereas the fluorescence (CellTiter-Blue®) and luminescence (CellTiter-Glo®) based assays resulted in high signals up to 40,000 cells per well. Data are shown as means and standard deviations of three independent experiments

## 2) Intact cell mass spectrometry

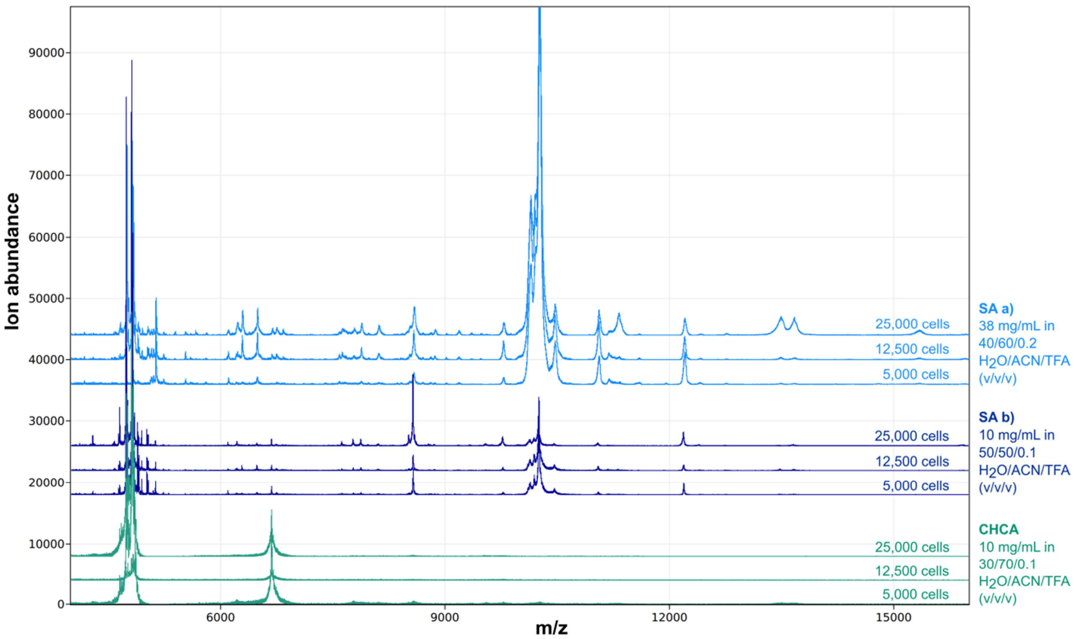


**Fig. S2** Intact cell MALDI-TOF MS analysis of different cell numbers of RTL-W1 and matrix solutions. A high concentrated sinapinic acid according to Munteanu et al. resulted in most significant peaks (SA a; Munteanu et al, 2012) compared to a lower concentrated SA matrix (SA b; Volta et al, 2012) or alpha-cyano-4-hydroxycinnamic acid matrix (CHCA, Dong et al, 2011). Mass spectra are shifted to allow discrimination of interspectral differences

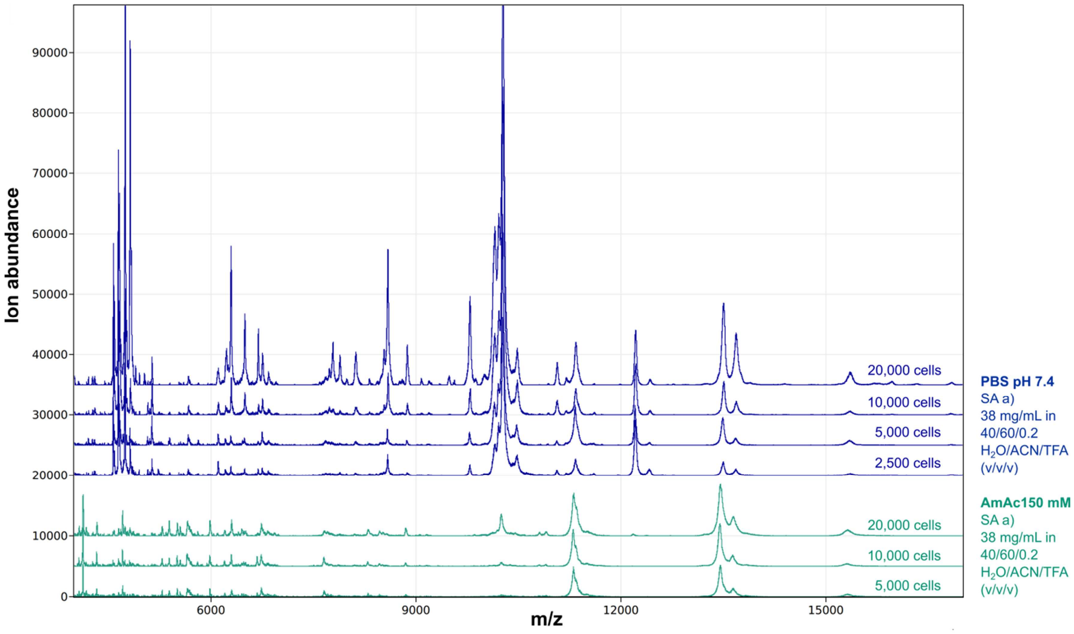


**Fig. S3** Intact cell MALDI-TOF MS analysis of RTL-W1 cells after washing with PBS respectively ammonium acetate (AmAc, 150 mM; Hanrieder et al, 2011). AmAc did not improve spectral quality
